# Roles of Ubiquitination and Deubiquitination in Regulating Dendritic Cell Maturation and Function

**DOI:** 10.3389/fimmu.2020.586613

**Published:** 2020-11-16

**Authors:** Bo Zhu, Lihua Zhu, Lin Xia, Yuyun Xiong, Qing Yin, Ke Rui

**Affiliations:** ^1^Department of Laboratory Medicine, Affiliated Hospital of Jiangsu University, Zhenjiang, China; ^2^International Genome Center, Jiangsu University, Zhenjiang, China; ^3^Department of Immunology, Jiangsu Key Laboratory of Laboratory Medicine, School of Medicine, Jiangsu University, Zhenjiang, China

**Keywords:** ubiquitination, dendritic cells, DC maturation, immune responses, post-translational modification

## Abstract

Dendritic cells (DCs) are specialized antigen-presenting cells that play a key role in immune homeostasis and the adaptive immune response. DC-induced immune tolerance or activation is strictly dependent on the distinct maturation stages and migration ability of DCs. Ubiquitination is a reversible protein post-translational modification process that has emerged as a crucial mechanism that regulates DC maturation and function. Recent studies have shown that ubiquitin enzymes, including E3 ubiquitin ligases and deubiquitinases (DUBs), are pivotal regulators of DC-mediated immune function and serve as potential targets for DC-based immunotherapy of immune-related disorders (e.g., autoimmune disease, infections, and tumors). In this review, we summarize the recent progress regarding the molecular mechanisms and function of ubiquitination in DC-mediated immune homeostasis and immune response.

## Introduction

Ubiquitination is a post-translational mechanism of protein modification that plays a crucial role in diverse biological processes by regulating the degradation and activity of substrate proteins (1, 2). The covalent conjugation of ubiquitin to lysine (K) residues of substrate proteins is mediated by an enzymatic reaction cascade. This process is catalyzed by the sequential activity of ubiquitin-activating (E1), ubiquitin-conjugating (E2), and ubiquitin-ligating (E3) enzymes (3). The substrate specificity of ubiquitination is primarily determined by E3 ubiquitin ligases, which recognize substrate proteins and catalyze the conjugation of ubiquitin to target proteins (3). The E3 ubiquitin ligases are a large, diverse group of proteins, characterized by one of several defining motifs. These have been historically grouped into two classes: the RING (really interesting new gene)–type E3 ubiquitin ligases and the HECT (homologous to the E6AP carboxyl terminus)–type E3 ubiquitin ligases (4). During the formation of polyubiquitin chains, the carboxyl-terminal glycine residue of ubiquitin is typically attached to an internal K residue or the amino-terminal methionine (M1) of another ubiquitin, which forms eight polyubiquitin chains, including K6, K11, K27, K29, K33, K48, K63, and M1 ubiquitin chains (5, 6). The type of polyubiquitin chains can influence the fate of the substrate, which exert distinct biological functions. For example, K48- and K11-linked polyubiquitin chains are usually involved in directing proteins for proteasome-dependent degradation, whereas K63-linked polyubiquitin chains have nonproteolytic functions, including the regulation of protein kinase activation and DNA damage repair (7). Ubiquitination is a reversible process that can be reversed by deubiquitination, which is performed by DUBs (8). DUBs can cleave conjugated ubiquitin chains from ubiquitinated substrates for signal transduction (8). Ubiquitination and deubiquitination play an essential role in the regulation of different aspects of immune function. The role of ubiquitination in lymphocyte biology has been reviewed elsewhere (6, 9–11). Therefore, this review will focus on dendritic cells (DCs).

DCs are specialized antigen-presenting cells that initiate and shape both the innate and adaptive immune responses (12). DCs arise from a hematopoietic lineage, which consist of developmentally and functionally distinct subsets. Under steady state conditions, DCs are customarily divided into conventional DCs (cDCs) and plasmacytoid DCs (pDCs). Moreover, cDC subsets comprise type 1 cDCs (cDC1s/CD8α^+^/CD103^+^DCs) and type 2 cDCs (cDC2s/CD11b^+^DCs), which are specialized at priming CD8^+^ and CD4^+^ T cells, respectively (13). Under inflammatory conditions, circulating Ly6C^hi^ monocytes directly differentiate into monocyte-derived DCs (MoDCs), which are induced by the inflammatory microenvironment and possess pro-inflammatory activity (13, 14). Recent studies have demonstrated that ubiquitination regulates DC-mediated immune homeostasis and adaptive immune responses. In this review, we describe the recent progress regarding the crucial role of ubiquitination in DC maturation and function (**Table 1**).

**Table 1:** Summary of the ubiquitin enzymes involved in regulating DCs maturation and function, as discussed in the text.

Ubiquitin enzyme	Type	Expression in DCs	Target	Action in DCs	Related diseases	References
MARCH1	E3 ligase	CD11c^+^DCs CD206^+^MoDCs	MHCII, CD86	Inhibits DCs phenotypic maturation Affects antigen presentation Affects DCs-mediated T cell activation Promotes MoDCs migration		(15–27)
WWP2	E3 ligase	DCs	MHCII	Inhibits DCs-mediated T cell activation	Salmonella infection	(28)
Hrd1	E3 ligase	CD11c^+^DCs	BLIMP	Impairs DCs-mediated priming of CD4^+^ T cells	EAE	(29)
A20	Deubiquitinase	CD11c^+^DCs	NEMO	Suppress DC maturation and cytokines production Maintains immune homeostasis Suppress T cells activation and differentiation Suppress B cells activation	systemic autoimmunity disease	(30–33)
PDLIM2 and MKRN2	E3 ligase	CD11c^+^DCs	p65	Suppress DC activation and cytokines production		(34–36)
Trabid	Deubiquitinase	CD11c^+^DCs	Jmjd2d	Promotes DCs-mediated Th1 and Th17 cells differentiation	EAE	(37)
OTUB1	Deubiquitinase	CD11c^+^DCs	UBC13	Promotes DCs cytokines production	Infection	(38)
sCYLD	Deubiquitinase	CD11c^+^DCs		Promotes DCs cytokines production Promotes DCs-mediated CD8^+^T cell response and NK cells activation	Infection	(39)
c-Cbl and Cbl-b	E3 ligase	BMDC	p105, p50	Promotes BMDC cytokines production Maintains immune homeostasis		(40–42)
CRL4 ^DCAF2^	Deubiquitinase	CD11c^+^DCs	NIK	Inhibits DCs cytokines production and Th17 cells differentiation Maintains immune homeostasis	Psoriasis EAE	(43)
UCH-L1	Deubiquitinase	CD11c^+^DCs	MHC I	Promotes DCs antigen cross-presentation	Listeria monocytogenes infection	(44)
CRL5 ASB2α	E3 ligase	CD11c^+^DCs	FLNa and FLNb	Promotes DCs migration		(45)

## Molecular Mechanisms of Ubiquitination-Mediated DC Maturation

### Regulation of MHCII and Costimulatory Molecules by Ubiquitination

Under physiological conditions, DCs maintain an immature or steady state to induce immune tolerance and maintain immune homeostasis (46). Upon toll-like receptor (TLR) stimulation, DCs undergo a transition from an immature to mature state and are accompanied by markedly upregulated membrane molecules, MHCII and costimulatory molecules, including CD80, CD86, and CD40 (13). Ubiquitination is an important mechanism that controls the surface expression and transport of MHCII by ubiquitin-dependent protein degradation in the lysosome (15, 16). The E3 ubiquitin ligase MARCH1, a member of the membrane-associated RING-CH (MARCH) family, ubiquitinates the β subunit of MHCII molecule *via* its cytoplasmic lysine (17). Apart from MHCII, MARCH1 also ubiquitinates CD86 in DCs by inducing intracellular degradation *via* the transmembrane domains (18, 19). Moreover, the transmembrane domains of MARCH1 interact but do not ubiquitinate CD83. CD83 competes with CD86 for interaction with MARCH1, thereby inhibiting CD86 ubiquitination and promoting the expression of CD86 on the surface of DCs (20). Overall, MARCH1 negatively regulates DC maturation by inducing the ubiquitin-dependent degradation of MHCII and CD86, and its expression is downregulated during DC maturation. In addition, ubiquitination can also affect DC maturation by regulating the level of MHCII transcription. Another E3 ubiquitin ligase, Hrd1, enhances MHCII gene transcription by promoting transcriptional repressor B lymphocyte-induced maturation protein (BLIMP) protein degradation through ubiquitination in DCs (29). A recent study shows that the NEDD4 family HECT E3 ubiquitin ligase WWP2 is required for SteD-dependent ubiquitination of mature MHCII during salmonella infection. As an adaptor, tumor suppressor protein TMEM127 binds to WWP2 and enables WWP2 to ubiquitinate MHCII, which contributes to the degradation of MHCII (28).

### Regulation of NF-κB Activation by Ubiquitination

**Figure 1:**
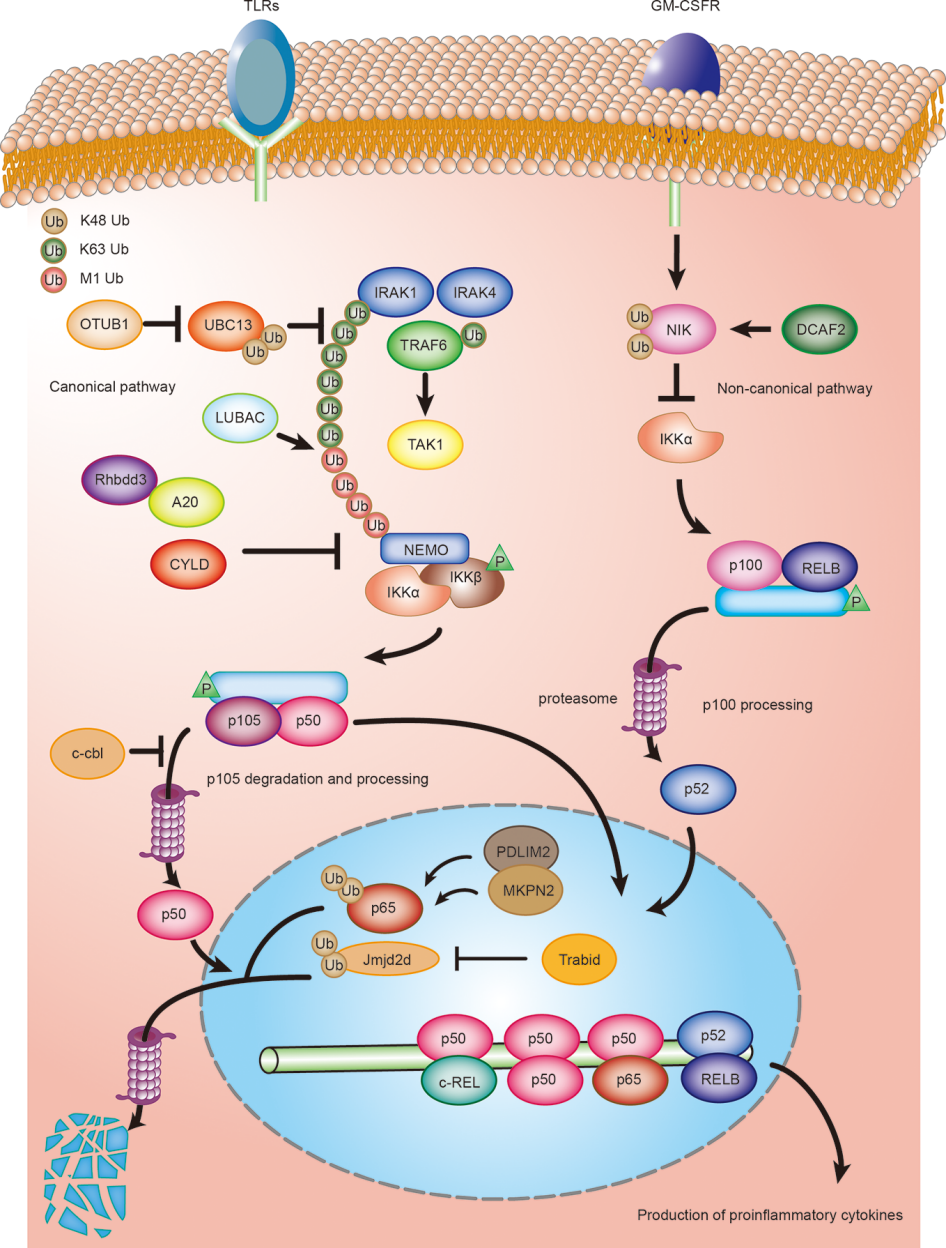
Ubiquitination regulating DCs activation and maturation by NF-κB signaling. Ubiquitination regulates both canonical and non-canonical NF-κB signaling in DCs. DCs are activated by toll-like ligands and inflammatory factors, such as GM-CSF, during an infection or inflammation. Activated DCs produce various cytokines, such as the proinflammatory cytokines IL-6, IL-12, and IL-23, which, in turn, regulate the differentiation of T cells. The deubiquitinase OTUB1 promotes canonical NF- κB activity by cleaving K48-linked polyubiquitination of UBC13. Rhbdd3 negatively regulates DC activation by recruiting A20 and facilitates A20-mediated deubiquitination of NEMO. CYLD inhibits activation of canonical NF-κB signaling by removing K63-linked polyubiquitin chains from NEMO. c-Cbl inhibits NF-κB signaling by stabilizing p105 and accumulating p50 via its RING domain, thus attenuating the recruitment of stimulatory NF-κB heterodimers and suppressing the activation of DCs. MKRN2 and PDLIM2 synergistically promote polyubiquitination and degradation of p65, thereby suppressing NF-κB-dependent activation of DCs. Trabid deubiquitinates and stabilizes Jmjd2d, a histone demethylase that removes transcriptionally repressive histone modifications, H3K9me2 and H3K9me3, from the Il12 and Il23 promoters to promote the recruitment of c-Rel, thereby facilitating the production of those cytokines in activated DCs. CRL4 DCAF2 negatively regulates IL-23 production in DCs by controlling NF-κB inducing kinase (NIK) stability and noncanonical NF-κB activation.

The canonical NF-κB signaling pathway is a key mediator of TLR-stimulated DC activation and functional maturation (47). Accumulating evidence suggests that ubiquitination plays a crucial role in the regulation of NF-κB signaling in both DC tolerance and activation. Several E3 ubiquitin ligases and DUBs in DCs regulate NF-κB activation by ubiquitination or deubiquitination of the NF-κB essential modulator NEMO (also known as IKK γ) and NF-κB subunits (p65 and c-Rel) (**Figure 1**). In general, TLR stimulation leads to the recruitment of the adaptor protein MyD88 and the kinases IRAK1 and IRAK4. Subsequent IRAKs now dissociate from the receptor complex and interact with E3 ubiquitin ligase TRAF6, which results in the K63 polyubiquitination of IRAK1/4 and TRAF6 itself by cooperating with the E2 ligases Ubc13 (48). As for TNFR stimulation, LUBAC (linear ubiquitin chain assembly complex) is required for full activation of NF-κB by the MyD88-dependent pathway. LUBAC complex extends K63-linked poly-ubiquitin chains with M1-linked poly-ubiquitin chains, resulting in recruitment and activation of the IKK complex through its adaptor NEMO, thereby liberating NF-κB transcription factors. In addition, three DUBs that play a crucial role in NF-κB signalling are OTULIN (OUT deubiquitinase with linear linkage specificity), CYLD (cylindromatosis), and A20 (48–50).

The ubiquitin-editing protein, A20, contains a deubiquitinase ovarian tumor (OTU) domain, which cleaves the K63-linked ubiquitin chains of NEMO, thereby inhibiting IKK and NF-κB activation (30, 51). It has been proposed that the rhomboid family protease, Rhbdd3, recruits A20 by binding K27-linked polyubiquitination on Lys302 of NEMO *via* the ubiquitin-binding-association (UBA) domain and facilitates A20-mediated deubiquitination of NEMO (31). An A20 or Rhbdd3 deficiency causes aberrant DC activation and increased production of the inflammatory cytokines, IL-12 and IL-6, in response to TLR ligands (31, 32). However, emerging evidence shows that the deubiquitinase activity of A20 is dispensable for NF-κB signaling (52). A20 negatively regulates TNF-induced NF-κB activation by binding and stabilizing linear ubiquitin chains (49, 50). Therefore, the precise molecular mechanisms that regulates TLR or TNF-induced NF-κB-dependent DC activation by A20 deserves further investigation. The nuclear protein, PDLIM2, as a nuclear E3 ubiquitin ligase, negatively regulates DC activation by promoting the p65 subunit of NF-κB polyubiquitination and proteasome-dependent degradation through its LIM (abnormal cell lineage 11-islet 1-mechanosensory abnormal 3) domain (34). The chaperone protein, HSP70, is required for the transport of the NF-κB-PDLIM2 complex to the proteasome (35). A deletion of PDLIM2 in DCs enhances the LPS-induced production of the proinﬂammatory cytokines, IL-6 and IL-12p40 (34). Interestingly, another study has reported that the E3 ubiquitin ligase, MKRN2, and PDLIM2 may synergistically promote polyubiquitination and p65 degradation, thereby suppressing NF-κB-dependent DC activation (34, 36). The E3 ubiquitin ligases, c-Cbl and Cbl-b, are two members of a highly evolutionary conserved family of Cbl proteins characterized as negative regulators of DC activation *in vitro*. c-Cbl inhibits the NF-κB pathway by stabilizing p105 and accumulating p50 *via* its RING domain, thereby attenuating the recruitment of stimulatory NF-κB heterodimers and suppressing the proinflammatory cytokine expression of IL-12 (40). Similarly, bone marrow–derived dendritic cells (BMDCs) from cbl-b deficient mice exhibit increased cytokine production (IL-1α, IL-6, and TNF-α) upon TLR stimulation. However, the molecular mechanisms by which Cbl-b is involved in DC activation remains largely unclear (41).

Jin et al. (37) reported that the deubiquitinase, Trabid, plays an essential role in mediating epigenetic regulation of TLR-induced expression of IL-12 and IL-23 in DCs. Another NF-κB family member, c-Rel, plays a key role in the TLR-induced expression of IL-12 and IL-23 (53, 54). Moreover, Trabid promotes the recruitment of c-Rel to the IL-12a, IL-12b, and IL-23a gene promoters by deubiquitinating and stabilizing the histone demethylase, Jmjd2d, which facilitates the production of these cytokines in activated DCs. Deubiquitination could also augment NF-κB- dependent DC activation by stabilizing the E2-conjugating enzyme. A recent study has demonstrated that OTUB1 promotes NF-κB activity in DCs by cleaving K48-linked polyubiquitination and increasing the stability of the E2-conjugating enzyme, UBC13 (38). UBC13 induces the activation of TGFβ-activated kinase 1 (TAK1) by ubiquitinating IL-1 receptor-associated kinase 1 (IRAK1) and TNF receptor-associated factor 6 (TRAF6), which is essential for NF-κB activity (55). In addition, a conditional deletion of OTUB1 in DCs impairs the TLR-induced activation of DCs and production of IL-12, IL-6, and TNF (38). Together, these data suggest a pivotal role for OTUB1 in driving NF-κB-dependent cytokine production by DCs. Another deubiquitinase, CYLD, is also an important regulator of NF-κB activity. Full-length CYLD inhibits activation of NF-κB signaling by removing K63-linked polyubiquitin chains from the signaling molecules, NEMO, TRAF2, and RIP1 (receptor-interacting protein 1) (56). However, the natural occurring short splice variant of CYLD (sCYLD), which lacks the TRAF2 and NEMO-binding sites, results in increased NF-κB activity (56). A conditional deletion of sCYLD in DCs augments the activation of CD8α^+^ DCs and the production of TNF, IL-10, and IL-12 in mice infected with Listeriosis. This suggests that this regulation depends on sCYLD‐mediated deubiquitination; however, the precise targets remain unclear (39).

Ubiquitination has also been found to play an important role in regulating the non-canonical NF-κB pathway involved in cytokine production by DCs. A recent study has revealed the E3 ubiquitin ligase, CRL4 ^DCAF2^, is involved in GM-CSF-induced expression of the inflammatory cytokine, IL-23. CRL4 ^DCAF2^ negatively regulates IL-23 production in DCs by controlling NF-κB–inducing kinase (NIK) stability and noncanonical NF-κB activation. Previous studies suggest that cellular inhibitor of apoptosis 1 and 2 (cIAP1/2) activation is mediated through its K63 ubiquitination by TRAF2 and promotes NIK degradation (57). Interestingly, CRL4 ^DCAF2^ directly promotes polyubiquitination and subsequent degradation of NIK independent regulation of cIAP activity. The DC-conditional deletion of DCAF2 enhances IL-23 production in DCs and promotes the development of psoriasis (43). Collectively, CRL4 ^DCAF2^ negatively regulates the production of the inflammatory cytokine, IL-23, in DCs by ubiquitinating NIK, implying a crucial role for CRL4 ^DCAF2^ on the non-canonical NF-κB-dependent cytokine production in DCs (**Figure 1**).

## Ubiquitination in DC-Mediated CD4 T Cell Responses

### Immune Homeostasis

It has been widely accepted that DC are inducers of central and peripheral tolerance, which plays a crucial role in maintaining immune homeostasis and blocking autoimmune responses (58, 59). DC-induced immune tolerance primarily depends on signaling pathway-mediated maturation, cytokine production, and immunomodulatory molecule expression of DCs. Ubiquitination plays a key role in the regulation of DC-induced immune tolerance. The E3 ubiquitin ligase, CRL4 ^DCAF2^, is critical for maintaining T cell homeostasis under physiological conditions. CRL4 ^DCAF2^ ablation in DCs impairs T cell homeostasis and causes spontaneous autoimmunity in aging mice, which is characterized by the increased frequency of Th17 effector T cells in the inguinal lymph nodes and elevated anti-nuclear antibodies in the serum (43). In addition, the deubiquitinase, A20, has also been well-characterized for this function. As discussed above, A20 inhibits the MyD88-NF-κB signaling axis in DCs to prevent spontaneous DC activation and activated T cell expansion (33). While an A20 deficiency in DCs has little effect on T cell development, the mice with a conditional A20 deficiency in DCs spontaneously developed systemic autoimmunity, including lymphocyte-dependent colitis, sero-negative ankylosing arthritis, and stereotypical conditions for human inflammatory bowel disease (IBD) (33). In addition, DCs lacking A20 exhibit increased co-stimulatory molecules expression and induce the generation of *in vivo* self-reactive Th1 and Th17 cells, which was accompanied by a high level of IL-6 secretion (33). Another study showed that the expression of A20 in DCs both preserves T cell homeostasis, and also directly inhibits B cell activation *in vitro* (30). These findings indicate that DCs require A20 to preserve immune homeostasis and suggest that A20 may function as a crucial checkpoint in the development of systemic autoimmunity, which provides a potential target for the therapeutic intervention of autoimmune diseases.

Rhbdd3, an upstream regulator of A20, and A20 binding and inhibitor of NF-κB-1 (ABIN-1) are also important to DC-induced immune tolerance. Rhbdd3 recruits A20 to promote NEMO deubiquitination and thereby negatively regulates DC activation. Rhbdd3 knockout mice spontaneously develop a systemic autoimmune phenotype, which is characterized by increased serum concentrations of immunoglobulins and antibodies and a reduced frequency of splenic Tregs, implying that Rhbdd3 is involved in controlling immune homeostasis (31). Similarly, as a ubiquitin-binding protein, ABIN-1 expression in DCs is necessary to preserve immune homeostasis (60). The DC-specific deletion of ABIN-1 exhibits excessive activation of NF-κB and MAPK signaling and produces more inflammatory cytokines (TNF, IL-6, IL-12, and IL-23) *in vitro*. Consequently, DC-conditional ABIN-1 deficient mice develop splenomegaly and lymphadenopathy by three to four months of age, characterized by an accumulation of myeloid cells and activated T lymphocytes (60).

While a deficiency of Cbl‐b alone exhibits no obvious effects on DC development and function (41), it has been reported that mice which a double ablation of c-Cbl and Cbl-b in DCs results in the development of severe liver inflammation (42). Cbl mutant mice display significantly increased cDC1 in the peripheral lymphoid organs and liver, which exhibits a hyperactivated phenotype and mediates the systemic activation of T and B cells. Further studies have shown that Cbls promote FMS-like tyrosine kinase 3 (FLT3) signaling *via* the ubiquitination of FLT3. This research suggests that both c-Cbl and Cbl-b–mediated ubiquitination hold the key for DC subset homeostasis and immune quiescence under steady‐state conditions, implying that c-Cbl and Cbl-b may have the potential to be therapeutic targets for the treatment of DC-mediated liver inflammation (42).

### Antigen Presentation

The MHCII molecule is crucial for the antigen presentation function of DCs by displaying antigenic peptides to CD4 T cells, which facilitates their activation, proliferation, and differentiation (61). MARCH1-mediated ubiquitination degradation of this molecule is an important mechanism that controls the surface expression of MHCII in DCs (15, 16). In agreement with this finding, MARCH1 expression is inversed to the level of surface MHCII expression during DC maturation (21). Thus, MARCH1 may negatively regulate the antigen presentation capability of DCs by reducing the surface expression of MHCII under steady state conditions (17). However, DCs deﬁcient in MHCII ubiquitination do not present more antigen, but exhibit a low Ag-presenting ability *in vivo* (22). Moreover, MHCII knock-in mice whose MHCII was not ubiquitinated show DC dysfunction similar to that of MARCH1 knockout mice (22). Therefore, MARCH1 both negatively regulate the surface expression of MHCII, and maintain DC function in the steady state *via* MHC II ubiquitination (15, 16, 22). Tregs are potent immune regulatory cells that upregulate MARCH1 expression in DCs by IL-10. This upregulation increases MHCII ubiquitination and reduces the surface expression of MHCII, which consequently results in impaired antigen presentation by DCs (20, 23, 24). WWP2 has also been shown to ubiquitinate and deplete the surface expression of mature MHCII, thereby suppressing DC-mediated T cell activation during Salmonella infection (28).

### CD4 T Cell Activation

In addition to affecting antigen presentation, MHCII ubiquitination can also regulate DC-mediated T-cell activation and development. However, the function of DC-mediated T-cell activation in MHCII ubiquitination appears to be highly complex. One study found that increased antigen presentation did not result in enhanced CD4 T cell activation (25). Moreover, MARCH1-deficient DCs have reduced, rather than increased, ability to activate naїve CD4^+^ T cells despite their higher level of surface MHCII expression (25). This finding may due to an excess accumulation of MHCII, which results in proteotoxicity and homeostatic disruption of the lipid raft and tetraspanin web in DCs (26). However, this has not been studied with mature naïve T cells, only for developing CD4+ thymocytes (26). In addition, MHCII ubiquitination is important for the development of thymic Tregs, but does not seem to affect the development of conventional T cells (62, 63). Ubiquitination also participates in DC-mediated T-cell activation by promoting MHCII gene transcription. The E3 ubiquitin ligase, Hrd1, increases MHCII expression through the ubiquitin-dependent degradation of BLIMP1. In addition, Hrd1 knockout mice exhibit impaired DC-mediated priming of CD4^+^ T cells and an attenuated autoimmune response (29).

As previously discussed, MARCH1 can regulate DC phenotypic maturation *via* ubiquitination-dependent degradation of the costimulatory molecule, CD86, thereby controlling DC-mediated T cell activation (18, 20). CD86 ubiquitination is an important mechanism of controlling the level of CD86 expression in DCs (19). DCs with ubiquitination-resistant mutant CD86 have greater T cell-activating abilities than that of DCs expressing wild-type CD86 *in vitro* (18). However, CD86 ubiquitination in DC-mediated T cell activation *in vivo* remains unknown.

### CD4 T Cell Differentiation

In addition to serving as APCs, DCs are also key regulators of CD4^+^ T cell differentiation by secreting different cytokines (64). Ubiquitination regulates cytokine production by DCs in response to different types of pathogens, which, in turn, guides the differentiation of CD4^+^ T cells to the generation of effector T cells, including the Th1 and Th17 subsets of helper T cells. It has been established that IL-12 and IL-23 overproduction are involved in the pathogenesis of autoimmune diseases through controlling the differentiation of naїve CD4^+^ T cells toward the Th1 and Th17 lineages, respectively (65, 66). The deubiquitinase, Trabid, in DCs promotes the generation of Th1 and Th17 cells and EAE pathogenesis through the epigenetic regulation of IL-12 and IL-23 expression (37). The culture medium from LPS-stimulated Trabid-deficient BMDCs decreases the differentiation of CD4^+^ T cells into Th1 and Th17 cells *in vitro* due to reduced IL-12 and IL-23 production (37). Furthermore, mice with a conditional deletion of Trabid in DCs are resistant to the induction of experimental autoimmune encephalomyelitis (EAE), which display a significantly lower frequency of Th1 and Th17 cells *in vivo*. However, T cells with a conditional Trabid deficiency have no defects in the *in vivo* production of Th1 and Th17 cells or *in vitro* T cell responses. These data demonstrate that Trabid mediates the production of IL-12 and IL-23 in DCs, thereby promoting Th1 and Th17 differentiation (37). In contrast to the positive role of Trabid in Th17 differentiation, E3 ubiquitin ligase, CRL4 ^DCAF2^, in DCs negatively regulates Th17 differentiation *in vivo* by limiting the production of IL-23 (43). Mechanistically, as discussed above, CRL4 ^DCAF2^ negatively regulates noncanonical NF-κB activation by inducing NIK ubiquitination and degradation, thereby inhibiting IL-23 production in DCs. A conditional DC deficiency of CRL4 ^DCAF2^ mice display substantially enhanced sensitivity to the induction EAE and have an increased frequency of Th17 inflammatory effector cells in the peripheral lymphoid organs (43).

A20 is another Th17-regulatory factor identified to be a deubiquitinase of NEMO, which regulates TLR- and TNF-triggered NF-κB activation (67). The DC-specific deletion of A20 promotes Th17 cell production both *in vivo* and *in vitro* through secreting high amounts of IL-23 (30). Intestinal DCs from DC-conditional A20 knockout mice have enhanced and biased abilities to promote inflammatory Th1 and Th17 cell differentiation, which is associated with lymphocyte-dependent colitis (68). In addition, Rhbdd3 recruits A20 to inhibit IL-6 production in DCs, and thus suppresses the generation of Th17 cells. Rhbdd3-deficient mice display increased susceptibility to Th17 cell-mediated colitis (31).

## Ubiquitination in DC-Mediated Cross-Priming of the CD8 T Cell Response

Antigen cross-presentation is crucial for an effective CD8^+^ T cell response in both intracellular bacterial infections and cancer (69). DCs have superior antigen cross-presentation ability to cross-prime CD8^+^ T cells both *in vivo* and *in vitro* (70, 71). Moreover, a recent study has revealed ubiquitination to be an important mechanism of regulating DC cross-presentation (44). The deubiquitinating enzyme, UCH-L1, has been shown to promote DC cross-presentation both *in vitro* and *in vivo*. In addition, UCH-L1 knockout mice infected with *listeria monocytogenes* exhibit impaired DC-mediated cross-priming of the CD8^+^ T cell response. Mechanistically, UCH-L1 promotes DC cross-presentation not by favoring antigen uptake or phagosome maturation, but by facilitating the recycling of MHC class I (MHC I) molecules. Reduced UCH-L1 expression in DCs impairs the colocalization of intracellular MHC I with late endosomal/lysosomal compartments, which is required for antigen cross-presentation. This data reveals that UCH-L1 expression in DCs plays a critical role in modulating DC-mediated cross-priming of the CD8^+^ T cell response, which facilitates the exploration of potential targets for therapeutic intervention against various infections and cancers. However, the substrate and E3 ubiquitin ligase for UCH-L1 in DCs remains to be defined.

## Ubiquitination in DC Migration

The migratory ability of DCs is key for the initiation of protective pro-inflammatory and tolerogenic immune responses (72). Apart from glycolytic metabolism and epigenetic pathways (73, 74), ubiquitination is also involved in the regulation of the migration of different DC subsets in health and disease (27, 45). A CRL5 complex, Cullin RING Ligase CRL5 ASB2α, which is highly expressed in immature DCs and down-regulated following DC maturation, which promotes DC migration by facilitating cell spreading and the formation of adhesive structures in immature DCs. Further research reveals that CRL5 ASB2α triggers polyubiquitylation and drives proteasome-mediated degradation of actin-binding protein filamins (FLNs), which are major organizers of the actin cytoskeleton (45). Interestingly, MARCH1-mediated MHCII ubiquitination is essential for the antigen presentation function of DCs but is also required for the migration of CD206-expressing monocyte-derived DCs (CD206^+^ MoDCs) to skin-draining lymph nodes (sdLNs) (27). The expression of IFN regulatory factor (IRF) 4 and C-C Motif Chemokine Receptor 7 (CCR7) play a pivotal role in controlling DC migration (72). CD206^+^ MoDCs from MARCH1 knockout mice exhibit MHCII overexpression and decreased IRF4 and CCR7 expression and have reduced migratory ability from the skin to sdLNs. Moreover, GM-CSF could restore CD206^+^ MoDC migration by promoting IRF4 expression both *in vitro* and *in vivo*. Collectively, these data suggest that the downregulation of MHCII by ubiquitination is crucial for the migration of CD206^+^ MoDCs to sdLNs in health and disease. While enhanced DC migration during early stages of the immune response is essential for the rapid induction of the immune response to eliminate invading pathogens, the timely termination of DC trafﬁcking at the late stage of the inﬂammatory response is required to prevent unwanted inﬂammation (74, 75). While current observations have primarily focused on the positive regulation of ubiquitination in DC migration, whether ubiquitination or deubiquitination can also function as a negative mediator that suppresses DC migration requires further investigation.

## Conclusion and Future Perspectives

In this review, ubiquitination was identified to play a critical role in DC maturation and function. To date, multiple E3 ubiquitin ligases and DUBs have been identified as key regulators of different physiological aspects of DCs, ranging from DC maturation to DC-mediated immune homeostasis and adaptive immune responses. These ubiquitin enzymes also play a key role in pathological processes mediated by DCs, indicating that they can be potential therapeutic targets for DC-based treatment of autoimmune diseases and cancer. The development of E3 ubiquitin ligase and DUB inhibitors with high selectivity should be considered as a promising immunotherapy approach. Bortezomib (or PS-341, trade name Velcade) is the first drug approved for targeting the UPS for the treatment of multiple myeloma (76). Several derivatives of bortezomib are at various stages of clinical trials for treatment of tumors (77). However, therapeutic approaches that selectively target the DCs ubiquitin-proteasome system in cancer and other diseases need to be further developed.

It has been well-established that ubiquitin enzymes are key players in the ubiquitination process; however, a major challenge in future studies will be to elucidate their underlying molecular mechanisms. For example, how each type of ubiquitin chain is generated, hydrolyzed, and recognized in DCs remains poorly understood. Whether acetylation and phosphorylation are involved in the translational modifications of ubiquitin remains unknown. Identification of the substrates of these enzymes is another challenge. Moreover, different DC subsets have both shared and unique functions; however, it remains largely unknown whether/how ubiquitin enzymes regulate the function of different DC subsets.

## Author Contributions

BZ and LZ drafted the manuscript. LX and YX discussed and revised the manuscript. QY and KR designed the study and revised the manuscript. All authors contributed to the article and approved the submitted version.

## Funding

This work was supported by the National Natural Science Foundation of China (Grant No. 81701612) and the Natural Science Foundation of Jiangsu (Grant No. BK20170563).

## Conflict of Interest

The authors declare that the research was conducted in the absence of any commercial or financial relationships that could be construed as a potential conflict of interest.

## References

[1] Deshaies RJ Joazeiro CA RING domain E3 ubiquitin ligases. Annu Rev Biochem (2009) 78:399–434. 10.1146/annurev.biochem.78.101807.093809 19489725

[2] Swatek KN Komander D Ubiquitin modifications. Cell Res (2016) 26(4):399–422. 10.1038/cr.2016.39 27012465 PMC4822133

[3] Hershko A Ciechanover A The ubiquitin system. Annu Rev Biochem (1998) 67:425–79. 10.1146/annurev.biochem.67.1.425 9759494

[4] Zheng N Shabek N Ubiquitin Ligases: Structure, Function, and Regulation. Annu Rev Biochem (2017) 86:129–57. 10.1146/annurev-biochem-060815-014922 28375744

[5] Berndsen CE Wolberger C New insights into ubiquitin E3 ligase mechanism. Nat Struct Mol Biol (2014) 21(4):301–7. 10.1038/nsmb.2780 24699078

[6] Hu H Sun SC Ubiquitin signaling in immune responses. Cell Res (2016) 26(4):457–83. 10.1038/cr.2016.40 PMC4822134 27012466

[7] Chen ZJ Sun LJ Nonproteolytic functions of ubiquitin in cell signaling. Mol Cell (2009) 33(3):275–86. 10.1016/j.molcel.2009.01.014 19217402

[8] Sun SC Deubiquitylation and regulation of the immune response. Nat Rev Immunol (2008) 8(7):501–11. 10.1038/nri2337 PMC5763493 18535581

[9] Ebner P Versteeg GA Ikeda F Ubiquitin enzymes in the regulation of immune responses. Crit Rev Biochem Mol Biol (2017) 52(4):425–60. 10.1080/10409238.2017.1325829 PMC5490640 28524749

[10] Yang XD Sun SC Deubiquitinases as pivotal regulators of T cell functions. Front Med (2018) 12(4):451–62. 10.1007/s11684-018-0651-y PMC6705128 30054854

[11] Wang A Zhu F Liang R Li D Li B Regulation of T cell differentiation and function by ubiquitin-specific proteases. Cell Immunol (2019) 340:103922. 10.1016/j.cellimm.2019.103922 31078284

[12] Ganguly D Haak S Sisirak V Reizis B The role of dendritic cells in autoimmunity. Nat Rev Immunol (2013) 13(8):566–77. 10.1038/nri3477 PMC4160805 23827956

[13] Merad M Sathe P Helft J Miller J Mortha A The dendritic cell lineage: ontogeny and function of dendritic cells and their subsets in the steady state and the inflamed setting. Annu Rev Immunol (2013) 31:563–604. 10.1146/annurev-immunol-020711-074950 23516985 PMC3853342

[14] Zhan Y Wu L Functional regulation of monocyte-derived dendritic cells by microRNAs. Protein Cell (2012) 3(7):497–507. 10.1007/s13238-012-0042-0 22773340 PMC4875395

[15] Shin JS Ebersold M Pypaert M Delamarre L Hartley A Mellman I Surface expression of MHC class II in dendritic cells is controlled by regulated ubiquitination. Nature (2006) 444(7115):115–8. 10.1038/nature05261 17051151

[16] van Niel G Wubbolts R Ten Broeke T Buschow SI Ossendorp FA Melief CJ Dendritic cells regulate exposure of MHC class II at their plasma membrane by oligoubiquitination. Immunity (2006) 25(6):885–94. 10.1016/j.immuni.2006.11.001 17174123

[17] Oh J Shin JS Molecular mechanism and cellular function of MHCII ubiquitination. Immunol Rev (2015) 266(1):134–44. 10.1111/imr.12303 PMC4677682 26085212

[18] Baravalle G Park H McSweeney M Ohmura-Hoshino M Matsuki Y Ishido S Ubiquitination of CD86 is a key mechanism in regulating antigen presentation by dendritic cells. J Immunol (2011) 187(6):2966–73. 10.4049/jimmunol.1101643 PMC4496154 21849678

[19] Corcoran K Jabbour M Bhagwandin C Deymier MJ Theisen DL Lybarger L Ubiquitin-mediated regulation of CD86 protein expression by the ubiquitin ligase membrane-associated RING-CH-1 (MARCH1). J Biol Chem (2011) 286(43):37168–80. 10.1074/jbc.M110.204040 PMC3199464 21896490

[20] Tze LE Horikawa K Domaschenz H Howard DR Roots CM Rigby RJ CD83 increases MHC II and CD86 on dendritic cells by opposing IL-10-driven MARCH1-mediated ubiquitination and degradation. J Exp Med (2011) 208(1):149–65. 10.1084/jem.20092203 PMC3023131 21220452

[21] Walseng E Furuta K Goldszmid RS Weih KA Sher A Roche PA Dendritic cell activation prevents MHC class II ubiquitination and promotes MHC class II survival regardless of the activation stimulus. J Biol Chem (2010) 285(53):41749–54. 10.1074/jbc.M110.157586 PMC3009902 21047782

[22] Ohmura-Hoshino M Matsuki Y Mito-Yoshida M Goto E Aoki-Kawasumi M Nakayama M Cutting edge: requirement of MARCH-I-mediated MHC II ubiquitination for the maintenance of conventional dendritic cells. J Immunol (2009) 183(11):6893–7. 10.4049/jimmunol.0902178 19917682

[23] Chattopadhyay G Shevach EM Antigen-specific induced T regulatory cells impair dendritic cell function via an IL-10/MARCH1-dependent mechanism. J Immunol (2013) 191(12):5875–84. 10.4049/jimmunol.1301693 PMC3858537 24218453

[24] Borges TJ Murakami N Machado FD Murshid A Lang BJ Lopes RL March1-dependent modulation of donor MHC II on CD103(+) dendritic cells mitigates alloimmunity. Nat Commun (2018) 9(1):3482. 10.1038/s41467-018-05572-z 30154416 PMC6113260

[25] Ishikawa R Kajikawa M Ishido S Loss of MHC II ubiquitination inhibits the activation and differentiation of CD4 T cells. Int Immunol (2014) 26(5):283–9. 10.1093/intimm/dxt066 24370470

[26] Oh J Perry JSA Pua H Irgens-Moller N Ishido S Hsieh CS MARCH1 protects the lipid raft and tetraspanin web from MHCII proteotoxicity in dendritic cells. J Cell Biol (2018) 217(4):1395–410. 10.1083/jcb.201611141 PMC5881489 29371232

[27] Majdoubi A Lee JS Balood M Sabourin A DeMontigny A Kishta OA Downregulation of MHC Class II by Ubiquitination Is Required for the Migration of CD206(+) Dendritic Cells to Skin-Draining Lymph Nodes. J Immunol (2019) 203(11):2887–98. 10.4049/jimmunol.1900593 31659013

[28] Alix E Godlee C Cerny O Blundell S Tocci R Matthews S The Tumour Suppressor TMEM127 Is a Nedd4-Family E3 Ligase Adaptor Required by Salmonella SteD to Ubiquitinate and Degrade MHC Class II Molecules. Cell Host Microbe (2020) 28(1):54–68 e7. 10.1016/j.chom.2020.04.024 32526160 PMC7342019

[29] Yang H Qiu Q Gao B Kong S Lin Z Fang D Hrd1-mediated BLIMP-1 ubiquitination promotes dendritic cell MHCII expression for CD4 T cell priming during inflammation. J Exp Med (2014) 211(12):2467–79. 10.1084/jem.20140283 PMC4235642 25366967

[30] Kool M van Loo G Waelput W De Prijck S Muskens F Sze M The ubiquitin-editing protein A20 prevents dendritic cell activation, recognition of apoptotic cells, and systemic autoimmunity. Immunity (2011) 35(1):82–96. 10.1016/j.immuni.2011.05.013 21723156

[31] Liu J Han C Xie B Wu Y Liu S Chen K Rhbdd3 controls autoimmunity by suppressing the production of IL-6 by dendritic cells via K27-linked ubiquitination of the regulator NEMO. Nat Immunol (2014) 15(7):612–22. 10.1038/ni.2898 24859449

[32] Xuan NT Wang X Nishanth G Waisman A Borucki K Isermann B A20 expression in dendritic cells protects mice from LPS-induced mortality. Eur J Immunol (2015) 45(3):818–28. 10.1002/eji.201444795 25472594

[33] Hammer GE Turer EE Taylor KE Fang CJ Advincula R Oshima S Expression of A20 by dendritic cells preserves immune homeostasis and prevents colitis and spondyloarthritis. Nat Immunol (2011) 12(12):1184–93. 10.1038/ni.2135 PMC3419270 22019834

[34] Tanaka T Grusby MJ Kaisho T PDLIM2-mediated termination of transcription factor NF-kappaB activation by intranuclear sequestration and degradation of the p65 subunit. Nat Immunol (2007) 8(6):584–91. 10.1038/ni1464 17468759

[35] Tanaka T Shibazaki A Ono R Kaisho T HSP70 mediates degradation of the p65 subunit of nuclear factor kappaB to inhibit inflammatory signaling. Sci Signal (2014) 7(356):ra119. 10.1126/scisignal.2005533 25515536

[36] Shin C Ito Y Ichikawa S Tokunaga M Sakata-Sogawa K Tanaka T MKRN2 is a novel ubiquitin E3 ligase for the p65 subunit of NF-kappaB and negatively regulates inflammatory responses. Sci Rep (2017) 7:46097. 10.1038/srep46097 28378844 PMC5380948

[37] Jin J Xie X Xiao Y Hu H Zou Q Cheng X Epigenetic regulation of the expression of Il12 and Il23 and autoimmune inflammation by the deubiquitinase Trabid. Nat Immunol (2016) 17(3):259–68. 10.1038/ni.3347 PMC4755875 26808229

[38] Mulas F Wang X Song S Nishanth G Yi W Brunn A The deubiquitinase OTUB1 augments NF-kappaB-dependent immune responses in dendritic cells in infection and inflammation by stabilizing UBC13. Cell Mol Immunol (2020). 10.1038/s41423-020-0362-6 PMC8167118 32024978

[39] Wurm R Just S Wang X Wex K Schmid U Blanchard N Protective dendritic cell responses against listeriosis induced by the short form of the deubiquitinating enzyme CYLD are inhibited by full-length CYLD. Eur J Immunol (2015) 45(5):1366–76. 10.1002/eji.201445116 25675948

[40] Chiou SH Shahi P Wagner RT Hu H Lapteva N Seethammagari M The E3 ligase c-Cbl regulates dendritic cell activation. EMBO Rep (2011) 12(9):971–9. 10.1038/embor.2011.143 PMC3166462 21799517

[41] Wallner S Lutz-Nicoladoni C Tripp CH Gastl G Baier G Penninger JM The role of the e3 ligase cbl-B in murine dendritic cells. PloS One (2013) 8(6):e65178. 10.1371/journal.pone.0065178 23762309 PMC3675148

[42] Li X Gong L Gu H Regulation of immune system development and function by Cbl-mediated ubiquitination. Immunol Rev (2019) 291(1):123–33. 10.1111/imr.12789 31402498

[43] Huang T Gao Z Zhang Y Fan K Wang F Li Y CRL4(DCAF2) negatively regulates IL-23 production in dendritic cells and limits the development of psoriasis. J Exp Med (2018) 215(8):1999–2017. 10.1084/jem.20180210 30018073 PMC6080916

[44] Reinicke AT Raczkowski F Muhlig M Schmucker P Lischke T Reichelt J Deubiquitinating Enzyme UCH-L1 Promotes Dendritic Cell Antigen Cross-Presentation by Favoring Recycling of MHC Class I Molecules. J Immunol (2019) 203(7):1730–42. 10.4049/jimmunol.1801133 31492742

[45] Lamsoul I Metais A Gouot E Heuze ML Lennon-Dumenil AM Moog-Lutz C ASB2alpha regulates migration of immature dendritic cells. Blood (2013) 122(4):533–41. 10.1182/blood-2012-11-466649 23632887

[46] Hammer GE Ma A Molecular control of steady-state dendritic cell maturation and immune homeostasis. Annu Rev Immunol (2013) 31:743–91. 10.1146/annurev-immunol-020711-074929 PMC4091962 23330953

[47] Kaisho T Tanaka T Turning NF-kappaB and IRFs on and off in DC. Trends Immunol (2008) 29(7):329–36. 10.1016/j.it.2008.03.005 18534908

[48] Griewahn L Koser A Maurer U Keeping Cell Death in Check: Ubiquitylation-Dependent Control of TNFR1 and TLR Signaling. Front Cell Dev Biol (2019) 7:117:117. 10.3389/fcell.2019.00117 31316982 PMC6609852

[49] Lork M Verhelst K Beyaert R CYLD, A20 and OTULIN deubiquitinases in NF-kappaB signaling and cell death: so similar, yet so different. Cell Death Differ (2017) 24(7):1172–83. 10.1038/cdd.2017.46 PMC5520167 28362430

[50] Spit M Rieser E Walczak H Linear ubiquitination at a glance. J Cell Sci (2019) 132(2). 10.1242/jcs.208512 30659056

[51] Harhaj EW Dixit VM Deubiquitinases in the regulation of NF-kappaB signaling. Cell Res (2011) 21(1):22–39. 10.1038/cr.2010.166 21119682 PMC3075605

[52] De A Dainichi T Rathinam CV Ghosh S The deubiquitinase activity of A20 is dispensable for NF-kappaB signaling. EMBO Rep (2014) 15(7):775–83. 10.15252/embr.201338305 PMC4196981 24878851

[53] Hilliard BA Mason N Xu L Sun J Lamhamedi-Cherradi SE Liou HC Critical roles of c-Rel in autoimmune inflammation and helper T cell differentiation. J Clin Invest (2002) 110(6):843–50. 10.1172/JCI15254 PMC151124 12235116

[54] Sanjabi S Hoffmann A Liou HC Baltimore D Smale ST Selective requirement for c-Rel during IL-12 P40 gene induction in macrophages. Proc Natl Acad Sci U S A (2000) 97(23):12705–10. 10.1073/pnas.230436397 PMC18828 11058167

[55] Ordureau A Smith H Windheim M Peggie M Carrick E Morrice N The IRAK-catalysed activation of the E3 ligase function of Pellino isoforms induces the Lys63-linked polyubiquitination of IRAK1. Biochem J (2008) 409(1):43–52. 10.1042/BJ20071365 17997719 PMC5791886

[56] Sun SC CYLD: a tumor suppressor deubiquitinase regulating NF-kappaB activation and diverse biological processes. Cell Death Differ (2010) 17(1):25–34. 10.1038/cdd.2009.43 19373246 PMC5848464

[57] Vallabhapurapu S Matsuzawa A Zhang W Tseng PH Keats JJ Wang H Nonredundant and complementary functions of TRAF2 and TRAF3 in a ubiquitination cascade that activates NIK-dependent alternative NF-kappaB signaling. Nat Immunol (2008) 9(12):1364–70. 10.1038/ni.1678 PMC2671996 18997792

[58] Iberg CA Hawiger D Natural and Induced Tolerogenic Dendritic Cells. J Immunol (2020) 204(4):733–44. 10.4049/jimmunol.1901121 PMC7006631 32015076

[59] Iberg CA Jones A Hawiger D Dendritic Cells As Inducers of Peripheral Tolerance. Trends Immunol (2017) 38(11):793–804. 10.1016/j.it.2017.07.007 28826942 PMC5669994

[60] Callahan JA Hammer GE Agelides A Duong BH Oshima S North J Cutting edge: ABIN-1 protects against psoriasis by restricting MyD88 signals in dendritic cells. J Immunol (2013) 191(2):535–9. 10.4049/jimmunol.1203335 PMC3702626 23785118

[61] Nakayama M Antigen Presentation by MHC-Dressed Cells. Front Immunol (2014) 5:672:672. 10.3389/fimmu.2014.00672 25601867 PMC4283639

[62] Oh J Wu N Baravalle G Cohn B Ma J Lo B MARCH1-mediated MHCII ubiquitination promotes dendritic cell selection of natural regulatory T cells. J Exp Med (2013) 210(6):1069–77. 10.1084/jem.20122695 PMC3674695 23712430

[63] Liu H Wilson KR Schriek P Macri C Blum AB Francis L Ubiquitination of MHC Class II Is Required for Development of Regulatory but Not Conventional CD4(+) T Cells. J Immunol (2020) 205(5):1207–16. 10.4049/jimmunol.1901328 32747505

[64] Walsh KP Mills KH Dendritic cells and other innate determinants of T helper cell polarisation. Trends Immunol (2013) 34(11):521–30. 10.1016/j.it.2013.07.006 23973621

[65] Gaffen SL Jain R Garg AV Cua DJ The IL-23-IL-17 immune axis: from mechanisms to therapeutic testing. Nat Rev Immunol (2014) 14(9):585–600. 10.1038/nri3707 25145755 PMC4281037

[66] Teng MW Bowman EP McElwee JJ Smyth MJ Casanova JL Cooper AM IL-12 and IL-23 cytokines: from discovery to targeted therapies for immune-mediated inflammatory diseases. Nat Med (2015) 21(7):719–29. 10.1038/nm.3895 26121196

[67] Zilberman-Rudenko J Shawver LM Wessel AW Luo Y Pelletier M Tsai WL Recruitment of A20 by the C-terminal domain of NEMO suppresses NF-kappaB activation and autoinflammatory disease. Proc Natl Acad Sci U S A (2016) 113(6):1612–7. 10.1073/pnas.1518163113 PMC4760784 26802121

[68] Liang J Huang HI Benzatti FP Karlsson AB Zhang JJ Youssef N Inflammatory Th1 and Th17 in the Intestine Are Each Driven by Functionally Specialized Dendritic Cells with Distinct Requirements for MyD88. Cell Rep (2016) 17(5):1330–43. 10.1016/j.celrep.2016.09.091 PMC5123685 27783947

[69] Kurts C Robinson BW Knolle PA Cross-priming in health and disease. Nat Rev Immunol (2010) 10(6):403–14. 10.1038/nri2780 20498667

[70] Ding Y Guo Z Liu Y Li X Zhang Q Xu X The lectin Siglec-G inhibits dendritic cell cross-presentation by impairing MHC class I-peptide complex formation. Nat Immunol (2016) 17(10):1167–75. 10.1038/ni.3535 27548433

[71] Ou P Wen L Liu X Huang J Huang X Su C Thioesterase PPT1 balances viral resistance and efficient T cell crosspriming in dendritic cells. J Exp Med (2019) 216(9):2091–112. 10.1084/jem.20190041 PMC6719428 31262842

[72] Worbs T Hammerschmidt SI Forster R Dendritic cell migration in health and disease. Nat Rev Immunol (2017) 17(1):30–48. 10.1038/nri.2016.116 27890914

[73] Guak H Al Habyan S Ma EH Aldossary H Al-Masri M Won SY Glycolytic metabolism is essential for CCR7 oligomerization and dendritic cell migration. Nat Commun (2018) 9(1):2463. 10.1038/s41467-018-04804-6 29941886 PMC6018630

[74] Liu J Zhang X Chen K Cheng Y Liu S Xia M CCR7 Chemokine Receptor-Inducible lnc-Dpf3 Restrains Dendritic Cell Migration by Inhibiting HIF-1alpha-Mediated Glycolysis. Immunity (2019) 50(3):600–15 e15. 10.1016/j.immuni.2019.01.021 30824325

[75] Lindquist RL Shakhar G Dudziak D Wardemann H Eisenreich T Dustin ML Visualizing dendritic cell networks in vivo. Nat Immunol (2004) 5(12):1243–50. 10.1038/ni1139 15543150

[76] Cheson BD Hematologic malignancies: new developments and future treatments. Semin Oncol (2002) 29(4 Suppl 13):33–45. 10.1053/sonc.2002.34878 12170431

[77] Popovic D Vucic D Dikic I Ubiquitination in disease pathogenesis and treatment. Nat Med (2014) 20(11):1242–53. 10.1038/nm.3739 25375928

